# Comments to “Radiofrequency Thermocoagulation as a Treatment for Hemifacial Spasm: Long-term Follow-up and Management of Recurrences”

**DOI:** 10.1007/s00701-024-06151-6

**Published:** 2024-06-10

**Authors:** Henry W. S. Schroeder

**Affiliations:** Department of Neurosurgery, University Medicine Greifswald, Greifswald, Germany

Paula Palomäki et al. published the paper “Radiofrequency Thermocoagulation as a Treatment for Hemifacial Spasm: Long-term Follow-up and Management of Recurrences” in this issue [5].

Thermocoagulation is known as a frequently applied treatment option for trigeminal neuralgia where the thinner pain fibers are destroyed while preserving the better myelinated sensory fibers. The application of thermocoagulation in hemifacial spasm is new to me.

The authors present 18 patients who underwent radiofrequency thermocoagulation (RFT) as a treatment for hemifacial spasm. 11 (61%) patients had repeated RFTs, and the mean number of RFTs per patient was 3.33. The mean follow-up was 5.54 years. The authors stated that patients were satisfied with the results after 87% of RFTs. Relief of the twitching of the face lasted 11.27 months (11.94 SD). Postoperative paresis lasted a mean of 6.47 months (6.80 SD). The authors conclude that RFT can be used to treat recurrences of HFS repeatedly and that RFT provides symptom relief for around 11 months.

The aim of treatment with RFT is to create a facial palsy. Although the authors stated that the palsy is usually moderate referred to as HB grad 3, after 12 procedures a HB 4, after 5 procedures a HB 5, and after 4 procedures even a total palsy (HB 6) occurred. I am surprised that the patients were nevertheless satisfied with the treatment result. In MVD, we do everything to avoid facial palsy. In our series (420 patients), we had 6 patients with immediate facial palsy and 25 with delayed facial nerve weakness. In the long-term, just 4 patients presented with HB grade 2 palsy [1]. In our experience, patients are not satisfied with the result when a facial palsy occurs.

Only 10 (56%) patients of the study had previously had MVD. Why was MVD not done as the primary procedure? If a frank explanation of the procedure, risks, and results would be done, I would expect more patients to choose MVD. Furthermore, delaying of MVD may result in deterioration of the intensity of spasms caused by progressing damage of the facial nerve at the compression site. We have seen paper-thin translucent nerves, which did not recover well because of delaying MVD. On the other side, MVD may cure the disease by removing the offending vessel from the facial nerve. The long-term success rate is around 90% [3]. RFT does provide relief of the spasms for 11.27 months with having a postoperative facial palsy for 6.47 months. I miss that the results of the study were not compared and discussed with the results of microvascular decompression (MVD).

Overall, it is proven by many reports that MVD is far superior in the long-term results with a very low rate of permanent facial palsy [4]. Therefore, it should be considered the procedure of choice if the clinical status of the patient allows general anesthesia. Even in recurrent cases, MVD should be considered first [2]. If the revision is not successful, RFT might be an option.

Henry W. S. Schroeder

Department of Neurosurgery, University Medicine Greifswald
